# Intelligent framework for diagnosis of frozen shoulder using cross sectional survey and case studies

**DOI:** 10.1186/s40064-016-3537-y

**Published:** 2016-10-21

**Authors:** Humaira Batool, M. Usman Akram, Fouzia Batool, Wasi Haider Butt

**Affiliations:** 1 National University of Sciences and Technology, Islamabad, Pakistan; 2 Riphah College of Rehabilitation Sciences, Riphah International University Islamabad, Islamabad, Pakistan

## Abstract

**Objectives:**

Frozen shoulder is a disease in which shoulder becomes stiff. Accurate diagnosis of frozen shoulder is helpful in providing economical and effective treatment for patients. This research provides the classification of unstructured data using data mining techniques. Prediction results are validated by K-fold cross-validation method. It also provides accurate diagnosis of frozen shoulder using Naïve Bayesian and Random Forest models. At the end results are presented by performance measure techniques.

**Methods:**

In this research, 145 respondents (patients) with a severe finding of frozen shoulder are included. They are selected on premise of (clinical) assessment confirmed after by MRI. This data is taken from the department of Orthopedics (Pakistan Institute of Medical Sciences Islamabad and Railway Hospital Rawalpindi) between September 2014 to November 2015. Frozen shoulder is categorized on the basis of MRI result. The predictor variables are taken from patient survey and patient reports, which consisted of 35+ variables. The outcome variable is coded into numeric system of “intact” and “no-intact”. The outcome variable is assigned into numeric code, 1 for “intact” and 0 for “no-intact”. “Intact” group is used as an indication that tissue is damaged badly and “no-intact” is classified as normal. Distribution of result is 110 patients for “Intact” group and 35 patients for “No-Intact” group (false positive rate was 24 %). In this research we have utilized two methods i.e. Naive Bayes and Random Forest. A statistics regression model (Logistic regression) to categorize frozen shoulder finding into “intact” and “no-intact” classes. In the end, we validated our results by Bayesian theorem. This gives a rough estimate about the probability of frozen shoulder.

**Results:**

In this research, our anticipated and predictive procedures gave better outcome as compared to statistical techniques. The specificity and sensitivity ratio of predicting a frozen shoulder are better in the Naïve Bayes as compared to Random Forest. In end the likelihood ratio results are used with Bayesian theorem for final evaluation of the results, from this we conclude predictive model is valid model for classification of frozen shoulder.

**Conclusions:**

We have used three predictive models in our study to classify frozen shoulder. Then we validated our predictive results by Bayesian theorem to give a rough estimate about the probability of occurrence of disease or not. This enhances the clinical decision making regarding frozen shoulder.

## Background

The tendons and bones that make up our shoulder joint are enclosed in a capsule of connective tissue. Whenever shoulder capsule become tightened and thick around the joint, the problem of frozen shoulder occurs (Crubbs 1993). Mostly frozen shoulder problem can be categorized into either primary also called unknown reason (idiopathic) or secondary (known reason) (Dias et al. 2005). Main signs and symptoms which lead to frozen shoulder are bad posture, post-operative (after surgery), stroke and patients suffering from diabetes (Mao et al. 1997). Frozen shoulder is more prone diabetics (20 %) than general population (2–5 %).Its ratio in insulin dependent patients are about 36 % (Afsar et al. 2014). Females are affected more than males (Manske and Prohaska 2010). Many treatments are available for frozen shoulder including both operative and non-operative. Operative procedures include manipulation under anesthesia and arthroscopic surgeries. Surgery reduces the severe complications of frozen shoulder. Non-operative includes pain management through different modalities (Transcutaneous electrical nerve stimulation, Short wave diathermy, Interferential therapy etc.), mobilization techniques, exercise plan and precautionary measures. All treatments just improve the functional time to recovery and increase the range of motion (Neviaser 1945). However, none of the treatment is authentic to totally eliminate the future chance of disease.

Main symptoms observed in frozen shoulder patients are pain, stiffness and loss of range of motion (Crubbs 1993). According to our knowledge, no work has been done on frozen shoulder using unstructured data (Ahmad et al. 2012). Only a few studies have been performed on clinical examinations and treatments with medical perspective, but no work has been done for the diagnosis of a frozen shoulder with respect to predictor values. The objective of this research is to develop reliable method for diagnosing frozen shoulder. Another significance of this study, is to analyze frozen shoulder intelligently by using unstructured data.

Physical examinations are generally considered low-cost process and results can directly be obtained at the time of the consultation. On the other hand, precision is based upon doctor’s knowledge and practice. Now a day’s frozen shoulder is diagnosed by clinical examination and imaging tests (Değerlendirmesi 2014). Firstly doctor diagnoses the problem by asking the patient to rotate the shoulder in a different direction. If physicians are uncertain about the problem, then formal test such as magnetic resonance imaging (MRI) can be carried out for diagnosis. Arthography is considered a standard test for the diagnosis of a frozen shoulder. It is having sensitivity 91 %, specificity 100 % and accuracy 92 %, but the test is an expensive and painful process (Ryu et al. 1993).

Better diagnosis and treatment plan for frozen shoulder are made through clinical findings. A lot of clinical examination methods have been developed to help in diagnosing frozen shoulder (Manske and Prohaska 2008). If patient is having limitations in hand elevation, then Apley scratch test is used for the diagnosis of disease. In Apley scratch test, patient is asked to put his/her arm above head and arrive at behind the neckline to touch his/her upper back. This test analyzes the rotation of upward, external and elevation (Anderson et al. 2011). During physical examination if the patient is having severe pain, then the physical assessment is marked positive. Previous researches showed that the ROM (range of motion) and Apley scratch test are good for diagnosing frozen shoulder (Woodward and Best 2000).

There is no existing fact, that any solitary check can diagnose frozen problem (Mitchell et al. 2005). Cost-effective treatment process is always dependent upon proper clinical assessment and diagnosis. The severity of the disease can be judged best by imaging tests as compared to clinical examination. According to the recent researches, if we make decision just on the basis of physical examination, it has sure chances that to give us false-positive ratio. Analysis made on the basis of physical examination always conflict with the imaging test. Due to this, we cannot make any decision just on the basis of clinical examination (Manske and Prohaska 2008; Bulgen et al. 1984; Clarke et al. 1974; Binder et al. 1984; Shaffer et al. 1992; Sharma et al. 1993).

This research provides a pathway towards accurate and correct diagnosis of frozen shoulder. The objective of this research is to develop reliable method for frozen shoulder. First milestone is to collect questionnaire from work related and chronic disease patients. Then on the basis of questionnaire and physical examination reports, we develop model which detect frozen shoulder category.

Identification of frozen problem by intelligence based framework is a new research area because previously it was based upon clinical examination. Clinical examination accuracy is dependent upon physician experience. Few case studies based researches have been done on clinical examination for the diagnosis of different shoulder problems but no research has been done on the correct diagnosis of shoulder problem by using intelligence framework. It may cause life time abnormality if not monitored well on time. So intelligence test should be used as a side assessment, for correct diagnosis of shoulder problem.

Data mining process is called as computational process. During computational process, software matches different patterns on the basis of logics. It classifies the huge datasets using different techniques like statistics and machine learning [ML] (Han et al. 2011; Bellazzi and Zupan 2008). This information discovery procedure turned out to be an accepted area to make inquires in diverse fields. It is being used in medical domain to discover patterns among medical variables. This also predicts disease outcomes using historical data (Bellazzi and Zupan 2008; Ramesh et al. 2004).

It is a difficult task for a doctor to properly diagnose and recommend treatment for some serious disease problems. In this context intelligent system model is considered as a useful procedure. We took information from different patients and then forecast a conclusion of interest (Bellazzi and Zupan 2008; Witten and Frank 2005). This will help us to take decision at a clinical level. Predictive methods of data mining are, Naïve Bayesian and Random Forest. These have been used most of the time to predict the conclusion of disease (Bellazzi and Zupan 2008; Griffith 2000). Some relevant studies used this concept; to provide prediction about how many patients are at high risk during anesthesia (Lin et al. 2011). Another study was on internal shoulder derangements (Oh et al. 1999).

Our main objective is to build up a procedure to diagnose frozen shoulder and to make decisions on the basis of physical information without depending on tests like MRI. Tests should only be used for detailed clinical hypothesis and only be recommended in severe and undiagnosed cases (Weinstein et al. 1980; Kassirer 1989; Pauker and Kassirer 1980). This study also used a predictive procedure of data mining and Chi Square test, to increase the precision of diagnosing frozen shoulder without physical examination. In this study we build a model and also made comparison among three predictive models (logistic regression, Random forest and Naïve Bayes) to categorize frozen shoulder groups on the basis of physical examination results, patient reports and survey.

### Proposed methodology

This research classifies the unstructured data using data mining techniques. It also used predictive models like Naïve Bayesian and Random forest to improve the diagnosis of frozen shoulder.

Methodology comprises of feasibility study, data gathering, preprocessing, and detecting predictor attributes. Then on the basis of training data, result was graded into different meaningful classification.

### Architecture

**Fig. 1:**
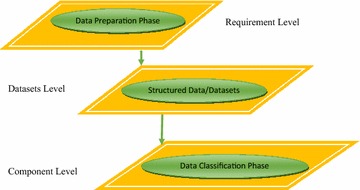
Architecture

This research used three tier architecture levels for analysis of disease. Figure 1 shows the architecture.

### Requirement level

This consists of collecting and finalizing data at single point. It also includes linkage of data with datasets level, to build an intelligent system for problem analysis. Requirement engineering [RE] process suggests the most common requirements according to the needs.

### Datasets level

This mainly deals with database. Database consists of datasets.

### Component level

This consists of framework to classify data.

### Block diagram of proposed system

**Fig. 2:**
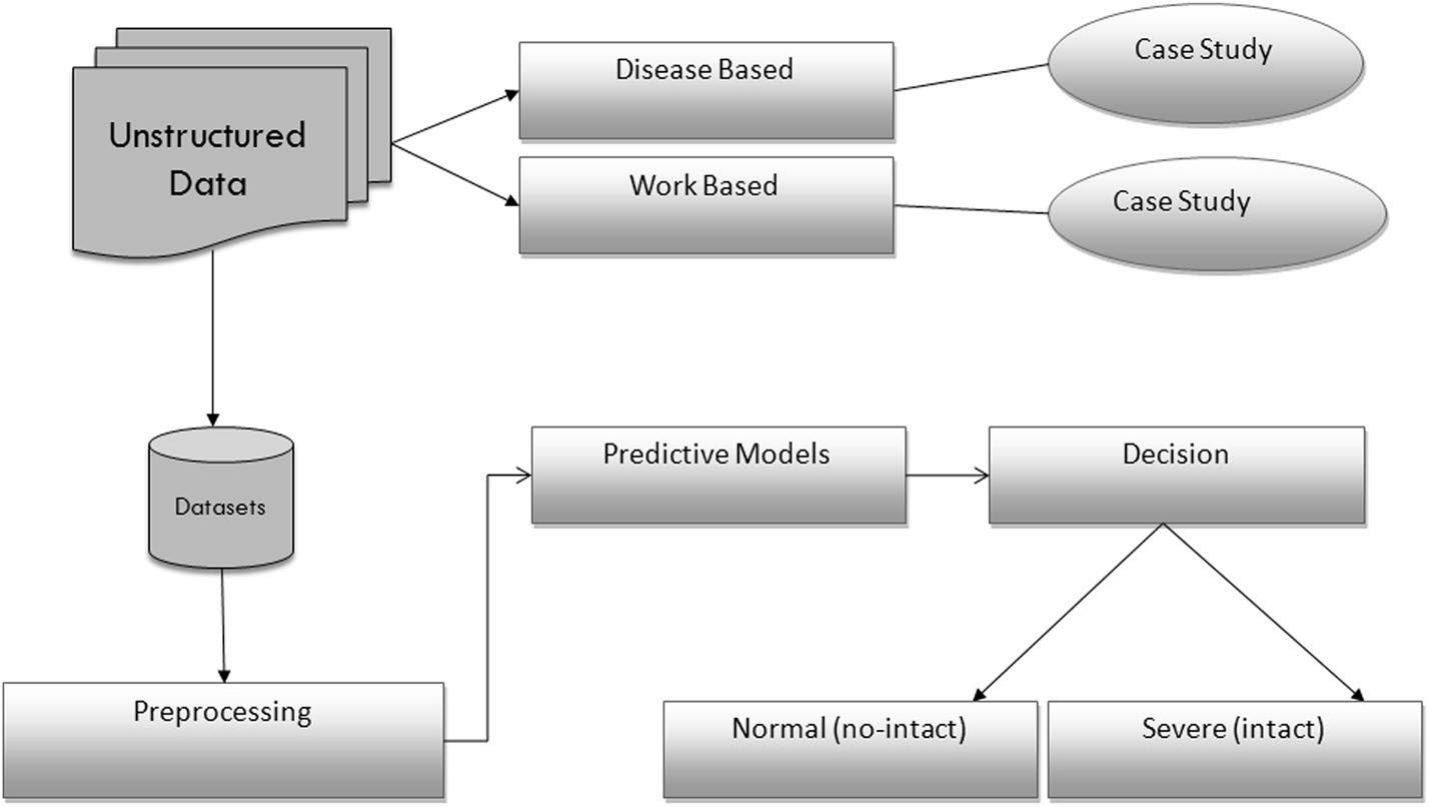
Block diagram of proposed system

Figure 2 shows the block diagram of the proposed system. The data we collected from patient reports and patient survey was in an unstructured form. Unstructured data mean raw data. Almost 80 % of medical data are available in an unstructured form like patient’s reports, lab report, and doctor review. We have collected history of different patients by case study method, for the purpose to get some predictor variables. All those case studies reveal that either the frozen shoulder problem occurred due to work related problem or either it is due to disease base problem. In the second level of architecture we converted unstructured data of patients into structured form by using software. Structure data was analyzed by the software. Then preprocessing was done on a datasets it includes feature extraction. Details about feature extraction method are mentioned in below section. In the third level of architecture we have applied different predictive model to classify unstructured data. This will also help in deciding either frozen shoulder patient classifies into intact class or no-intact class.

### Requirement engineering process

In our research study, we have used a well known requirement engineering process model for data preparation. Requirement engineering process is called as “The process to gather the requirements from client analyze and document them”. Data preparation output would be used for predicting result accurately and intelligently. Client involvement may be a major consideration in the development of most successful systems. The main purpose of using requirement engineering process in our study is that, the most difficult phase is to collect patient’s information and user involvement for finalizing all data at some single point. So far it is largely unexplored. Model defines the approaches and methods used to deal with difficulties.

Requirement engineering is a four step process, which includes feasibility study, requirement gathering, requirement specification, and requirement validation. Each stage has its specific tasks and outputs.

The emphasis of first step is on getting better understanding of project objectives to establish better plans to achieve business and intelligent goals. Feasibility study is an analysis of the importance of an idea. Conducting a feasibility study is a good practice to gives focuses to the project and outline alternatives. Next step includes data collection from the patient and doctors. It involves communicate with the patient and doctors to know their views about disease and to identify important features for survey. Data understanding is achieved by categorizing data into work based, disease based. After finalizing the requirements next phase of the process is requirement specification, requirement specification is same like blueprints. Framework selection according to domain is an art which can easily tie all internal factors of requirements. Framework design usually utilize the most time of the project. When business goals are settled then next step is requirement validation. Requirement validation step involves to check whether all important points are covered correctly and easily validate through alternatives. It also confirms that whether all domain requirements are accurately adjusted in a document or not.

**Fig. 3:**
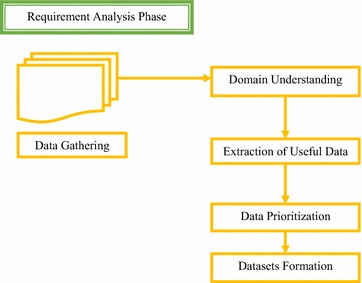
Requirement analysis phase

This study was conducted with the collaboration of Pakistan Institute of Medical Science Islamabad and Railway Hospital Rawalpindi, Pakistan. A real report based data of frozen shoulder patients was used. In this study, original data set is used that was obtained from Hospitals. For the purpose of domain understating we covered different case studies. This composed data was unstructured patient’s records. First we transformed the unstructured reports/survey into planned order and then picked out important variables. By hands mean, we have selected some hidden predictive variables from the patient reports, which were obtained from the information doctor has taken during the subjective examination or history taking. We designed a patient survey with the assistance of therapeutic specialists (practitioners).Extraction of meaningful information from the survey was done very carefully. The requirement engineering process was used to decide whether the extracted attributes are sufficient and contains all the meaningful attributes or still extra information is required to get important variables. The mining (extraction) of variables is key benefit to get the knowledge of problem field plus it would assist additionally within the study. After the categorization of data, datasets was formed. Requirement analysis steps are shown in a Fig. 3.

### Significance of irregular (unstructured) data

In 1998, Merrill Lynch declared that a normal 80 % of all business information has it source in unstructured form (Murdoch and Detsky 2013). Mostly larger part of business information should came from unstructured source. As indicated by another source (Murdoch and Detsky 2013), around 80 % of the medical information that might be patient’s reports is in irregular form. Once the unorganized information is organized and put in database is called planned data. The planned information is easily comprehensible by computers and is used by framework for problem analysis.

### Datasets level

We have collected meaningful information from last level which would be helpful for decision. We designed the datasets to grasp the collected variables. The patient survey data set contained different variables which were categorical, nominal and scale data. The description and values of patient’s survey variables are stated in Table 1. The description and values of patient’s reports variables are stated in Table 2.

**Table 1:** Variable list of patient survey

Variables	Type	Coding
*Outcome*		
Frozen shoulder	Nominal	2 codes (1 for Yes, 0 for No)
*Predictor*		
Age	Ordinal	3 codes (20–45, 46–60, ≥ 61)
Gender	Nominal	2 codes (1 for male, 0 for female)
Occupation	Nominal	4 codes
Hand	Nominal	2 codes (1 for right, 2 for left)
BMI	Ordinal	3 codes
Conditions	Nominal	14 codes
Medications	Nominal	11 codes
Smoke	Nominal	2 codes (1 for yes, 0 for no)
Stress	Ordinal	3 codes
Problem	Nominal	6 codes
Complaint	Nominal	6 codes
Shoulder	Nominal	2 codes (1 for right, 2 for left)
Sharp pain	Nominal	2 codess (1 for yes, 0 for no)
Dull Pain	Nominal	2 codes (1 for yes, 0 for no)
Aching	Nominal	2 codes (1 for yes, 0 for no)
Throbbing	Nominal	2 codes (1 for yes, 0 for no)
Numbness	Nominal	2 codes (1 for yes, 0 for no)
Shooting	Nominal	2 codes (1 for yes, 0 for no)
Burning	Nominal	2 codes (1 for yes, 0 for no)
Pain	Nominal	4 codes
Pain score	Nominal	2 codes
Wake	Nominal	2 codes (1 for yes, 0 for no)
Before	Nominal	2 codes (1 for yes, 0 for no)
Dislocated	Nominal	2 codes (1 for yes, 0 for no)
Injection	Nominal	2 codes (1 for yes, 0 for no)
Surgery	Nominal	2 codes (1 for yes, 0 for no)
Medicine	Nominal	2 codes (1 for yes, 0 for no)
Interfere	Nominal	2 codes (1 for yes, 0 for no)
Activity	Nominal	2 codes (1 for yes, 0 for no)
Lifting	Nominal	2 codes (1 for yes, 0 for no)
Behind	Nominal	2 codes (1 for yes, 0 for no)
Sports	Nominal	2 codes (1 for yes, 0 for no)
Decrease	Nominal	4 codes
MRI	Nominal	2 codes (1 for yes, 0 for no)
Repetitive	Nominal	2 codes (1 for yes, 0 for no)
Hours	Nominal	5 codes
Improvement	Nominal	3 codes
Killers	Nominal	2 codes (1 for yes, 0 for No)
Trauma	Nominal	2 codes (1 for yes, 0 for No)
Working	Nominal	2 codes (1 for yes, 0 for No)
Dominant	Nominal	5 codes
History	Nominal	10 codes

**Table 2:** Variable list of patient report

Variables	Type	Coding
Active range of motion	Nominal	2 codes (1 for positive, 0 for negative)
Passive range of motion	Nominal	2 codes (1 for positive, 0 for negative)
Internal rotation	Nominal	2 codes (1 for positive, 0 for negative)
External rotation	Nominal	2 codes (1 for positive, 0 for negative)
Flexion	Nominal	2 codes (1 for positive, 0 for negative)
Abduction	Nominal	2 codes (1 for positive, 0 for negative)
Apley’s scratch test	Nominal	2 codes (1 for positive, 0 for negative)

### Data collection procedure

In this research, 145 respondents (patients) with severe symptoms of frozen shoulder were included. They were selected on the premise of (clinical) assessment confirmed after by MRI. This data was taken from the department of Orthopedics (Pakistan Institute of Medical Sciences Islamabad and Railway Hospital Rawalpindi) between September 2014 to November 2015. Frozen shoulder was categorize on the basis of MRI result.MRI result was used a standard.

The result variable was coded into numeric system of “intact” and “no-intact”. “Intact” group incorporated that tissue has been damaged badly and “no-intact” was classified as normal tissue. The result distribution variable was 110 for “Intact” and 35 patients for “No-Intact” (False positive rate of 24 %). The judgment variable was the clinical results, which consist of 35+ variables. Apley’s Scratch Test and Resisted Tests (ROM) are common provocative tests in diagnosing frozen shoulder (Woodward and Best 2000). These two tests were utilized as screening/diagnostic tests in the analysis models. In this research, we have utilized three mining methods to categorize frozen shoulder (intact and no-intact classes).it includes, Naive bayes and the Random Forest and a statistics regression model (logistic regression). Dataset consisted of surveyed questions and reports. It contained all the data on the subject of abnormalities and their relation, as it is used for assessment and association purposes. However, for this study there was no availability of dataset either locally or globally. Due to this, we have collected data from different hospitals by survey method. The assessment of proposed framework of frozen shoulder patients was performed on a locally gathered dataset.

## Methods

Precision in medical systems is extremely significant. We have used three tier architecture. It includes requirement level, datasets level and component level. First two levels have already been explained in the previous section. Component level includes feature extraction and classification techniques. The study also helps in comparison with different classification techniques.

This paper includes proposed framework results and details of dataset. Comparison with the other techniques is also included for the validation of proposed system. We used statistics regression model (logistic regression) to assess the outcomes (Lin et al. 2010; Bagley et al. 2001). The result of this model is then compared with other two Classification Techniques (which are Nave Bayes and Random Forest). We have used cross validation techniques for performance authentication. Verification was done by comparing the accuracy of classification models.

### Feature selection

In this step unrelated variables and unnecessary information was eliminated. At the start numbers of variables were 35 + , extracted from the survey and reports. A few of the variables were unnecessary and was not helpful in decision making. Those variables were removed by feature selction method. Data preprocessing includes feature selection, which helps in decreasing the unrelated data and improves the learning effectiveness. This also increases the accuracy of models. After this procedure, resulted variables are those variables that are helpful for identification of purpose and also produce high accuracy for classification models. Feature extraction was done by *T* TEST technique and significance probability value was taken as 0.05.

Most of the research questions have been validated through hypothesis. The research questions are for our knowledge and therefore the statistics that comes hereafter. Whenever, we have created hypothesis test in statistics, a P value always help us to conclude the importance of our outcome. Basically hypothesis tests are used to check the strength of a claim that is made about a community. This claim is known as the null hypothesis. After the outcome, if the null hypothesis is inaccurate then we would accept the other hypothesis as true. In statistics, P value is a range among Zero and One and translated in the subsequent way:

- After study if results show small P value (≤0.05), then we can easily reject the null hypothesis because smallest P value shows strong evidence against the null hypothesis.
- After study if results show large P value (>0.05), then we cannot reject the null hypothesis because large P value shows weak evidence against the null hypothesis.
- After study if results show P values near the cutoff (0.05), then we could go either way because results are on margin.

### Component level (classification phase)

In this section, we would explain in detail about the component steps. It is used to categorize the data. We will compare different classification techniques. At the end, conclusion can be made on the basis of comparison results.

### Training and testing procedure

Generally the error is related with randomness results in biasing the estimation. Cross-validation is regularly applied to minimize the error. The power of a prediction model to generalize unseen data can be frequently validated by K-fold cross validation method. All sampling data is divided into K equal size subsamples randomly, by K-fold cross-validation procedure strategy. We have used 50 % data for testing purpose and 50 % data for training purpose. The both method (training and testing) is then repeated K time. In this study, K-fold cross-validation number ten was chosen in light of the fact that numerous researches have demonstrated that ten as an ideal validation number. In the tenfold cross-approval, the method was repeated ten times with totally dissimilar training and testing datasets (Kohavi 1995; Bengio and Grandvalet 2004; Breiman et al. 1984).

We have additionally checked the error of different predictive models by performance measures method during comparative analysis (Delen et al. 2005).

## Results

We have collected data from 145 frozen shoulder patients by survey and patient’s real reports. Table 3 shows the demographic data of the patients. The majority patients were Female (71 %); the age ranges were in between 46–60 years (44.8 %).

**Table 3:** Demographic Data of the 145 Patients

Description	Characteristics	Frequency	Percentage
Age	20–45	48	33.1
	46–60	65	44.8
	≥61	32	22.1
Gender	Male	42	29.0
	Female	103	71.0
Occupation	Housewife	63	43.4
	Employee	65	44.8
	Un-employee	02	1.4
	Retired	15	10.3
Hand	Right	126	86.9
	Left	19	13.1
BMI	Overweight	17	11.7
	Underweight	48	33.1
	Normal weight	80	55.2
Conditions	Arthritis	01	0.7
	Diabetes	07	4.8
	Depression	01	0.7
	Other	02	1.4
	Diabetes-depression	23	15.9
	HBP-diabetes-depression	55	37.9
	Difficulty sleeping-HBP-diabetes-depression	52	35.9
	Arthritis-HBP	02	1.4
	HBP-diabetes-depression-heart Disease	02	1.4
Smoke	Yes	27	18.6
	No	118	81.4
Stress	High	84	57.9
	Medium	54	37.2
	Low	07	4.8
Shoulder	Right	57	39.3
	Left	88	60.7
Wake	Yes	125	86.2
	No	20	13.8
Problem	Pain	30	20.7
	Stiffness	10	6.9
	Dislocation	02	1.4
	Pain-stiffness	78	53.8
	Pain-stiffness-dislocation	25	17.2
Complain	Injury	16	11.0
	Work-based	63	43.4
	Disease-based	42	29.0
	Surgery	01	0.7
	Injury-work-based	05	3.4
	Injury-disease-based	18	12.4
Pain	Constant	33	22.8
	Frequent	52	35.9
	Occasional	57	39.3
	Intermittent	03	2.1
Pain Score	Average	60	41.4
	Worst	85	58.6
Before	Yes	40	27.6
	No	105	72.4
Dislocated	Yes	38	26.2
	No	107	73.8
Injection	Yes	52	35.9
	No	93	64.1
Surgery	Yes	30	20.7
	No	115	79.3
Medicine	Yes	116	80
	No	29	20
Interfere	Yes	140	96.6
	No	05	3.4
Activity	Yes	121	83.4
	No	24	16.6
Lifting	Yes	139	95.9
	No	06	4.1
Behind	Yes	140	96.6
	No	05	3.4
Sports	Yes	30	20.7
	No	115	79.3
Decrease	Heat	81	55.9
	Ice	01	0.7
	Rest	09	6.2
	Rest-heat	54	37.2
MRI	Yes	110	75.9
	No	35	24.1
Repetitive	Yes	112	77.2
	No	33	22.8
Killers	Yes	112	77.2
	No	33	22.8
Trauma	Yes	18	12.4
	No	127	87.6

Majority respondents were employee (44.8 %) and housewife. Majority of the patients dominant hand was right (86.9 %) and their body mass was normal (55.2 %). Majority of the patients were suffering from HBP-diabetes-depression (37.9 %).Stress level in majority of patients was high due to occupation (57.9 %). Left shoulder was involved in majority of the respondent (60.7 %).Those patients wake at night with pain ratio was (86.2 %); (53.8 %) respondents were having main complaint of the pain-stiffness. History of complain; due to work related activities were present in (43.4 %) patients. Origin of pain was sudden in (39.3 %) respondents and nearly 58.6 % of patients rated their pain as worst. The ratio of patients with no such complaint before was (72.4 %). Majority was not having any previous history of dislocation, injection in a shoulder and before history of surgery and (80 %) patients were on medication. Majority respondents told that pain interferes with their routine work (96.6 %) and also with their sports activities. (55.9 %) patients told that after heat therapy they feel better but that is just for some time. Majority said that repetitive lifting loads at their work place are main cause of pain and almost (77.2 %) were using pain killers for musculoskeletal problems (77.2 %).

Clinical examination is being done mostly on the basis of Active range of motion, internal rotation, flexion and Apley’s scratch. These are considered as standard interpreter attributes to decide the frozen shoulder. The proportions of positive finding were 97.2, 89.0, 93.1 and 89.0 % respectively. Respondent, were having negative passive range of motion, external rotation were positive 91.7 %, Abduction test were positive 42.8 as shown in Table 4. The assessment attributes also included different kind of pain types (which are sharp, aching, throbbing, numbness, shooting, burning pain) which were then coded into two responses. The response having sharp pain was (82.1 %) and percentage of aching pain was (64.8 %).The throbbing pain was (95.9 %). Some patients had numbness (80 %), burning (77.2 %) and shooting ratio was 91.0 %.

**Table 4:** Symptoms related data of patients

Description	Characteristics	Frequency	Percentage
Sharp pain	Yes	119	82.1
	No	26	17.9
Aching	Yes	94	64.8
	No	51	35.2
Throbbing	Yes	139	95.9
	No	06	04.1
Numbness	Yes	116	80
	No	29	20
Shooting	Yes	13	9.0
	No	132	91.0
Burning	Yes	112	77.2
	No	33	22.8
Active range of motion	Positive	141	97.2
	Negative	04	02.8
Passive range of motion	Positive	48	33.1
	Negative	97	66.9
Internal rotation	Positive	129	89.0
	Negative	16	11.0
External rotation	Positive	133	91.7
	Negative	12	8.3
Flexion	Positive	135	93.1
	Negative	10	6.9
Abduction	Positive	62	42.8
	Negative	83	57.2
Apley’s scratch test	Positive	129	89.0
	Negative	16	11.0

The P value was used to look at the valuable attribute between the “intact” and “no-intact” class. Table 5 shows the valuable attribute between Intact and No-Intact class. The asterisk (*) code in a last column shows the significance of each predictor variable. Variables with **** code are the most significant ones and all others variable without any * has no significance association. Along with MRI report, we have assigned class labels to every record.

**Table 5:** Valuable variables between intact and no-intact group

S. no	Variable	P value	Code	S. no	Variable	P value	Code
01	Age	0.670		21	Before	0.342	
02	Gender	0.001	****	22	Dislocated	0.434	
03	Occupation	0.002	***	23	Injections	0.089	*
04	Hand	0.386		24	Surgery	0.958	
05	BMI	0.896		25	Medicines	0.037	**
06	Conditions	0.894		26	Interfere	0.933	
07	Smoke	0.590		27	Activity	0.465	
08	Stress	0.147		28	Lifting	0.928	
09	Problem	0.003	***	29	Behind	0.933	
10	Complaint	0.680		30	Sports	0.390	
11	Shoulder	0.991		31	Repetitive	0.004	***
12	Sharp	0.297		32	Killers	0.010	**
13	Ache	0.787		33	Trauma	0.530	
14	Throbbing	0	****	34	Active ROM	0.435	
15	Numbness	0.001	****	35	Passive ROM	0.486	
16	Shooting	0	****	36	Abduction	0.446	
17	Burning	0	****	37	Internal	0	****
18	Pain	0.008	***	38	External	0.000	****
19	Score	0.171		39	Flexion	0.000	****
20	Wake	0.009	***	40	Apley	0	****

Probability method evaluated the significance of an attribute by computing the worth of P value measurement. It uses ranker search technique with threshold P ≤ 0.05. This search method gives weight and P value by their individual evaluations. Mostly P value was used to check the similarity between the Intact and No-Intact groups of each variable. Those variables which have mentioned in Table 5 with Starric (**) codes, are selected due to their P value less than 0.05. Four (****) code variables are more significant follow by three codes, two codes and one code. One (*) code is least significant due to having more P value among the other significant variables. The performance of the proposed system is evaluated on significant variables. The effective attributes are shown in Table 5.

Firstly we separated variables against the code. According to the coding scheme, we have separately applied model on different codes and then judged the accuracy of different variable codes. We have also validated our results and calculated the accuracy by the formula given in Eq. 1.

1 $$ \left( {{{{\text{Valid number of predictions}} * 1.00} \mathord{\left/ {\vphantom {{{\text{Valid number of predictions}} * 1.00} {\text{Total number of patients}}}} \right. \kern-0pt} {\text{Total number of patients}}}} \right) * 100 $$

### Models comparative analysis

We have used different prediction models for this study. Firstly, we have analyzed the data by logistic regression model. After model implementation, we have used two other classification techniques for the purpose of analysis and comparison with Logistic Regression. The following results were obtained: LR model classified data with 96.55 % accuracy during training of model. LR model gave 95.10 % accuracy during cross validation. We have applied the same steps on Naïve Bayes and Random Forest. Table 6 results show that Random Forest categorizes the data 94.48 % while training, it gives 89.81 % accuracy during cross validation time. For Naïve Bayes we achieved 99.31 % accuracy during training of model, it gives 99.29 % accuracy during cross validation. Overall results show that, Naïve Bayes model performs most excellent on our data along with other procedures and techniques. Table 6 shows the classification models results.

**Table 6:** Classification models results with prediction performance

Algorithm	Precision	AUC	Specificity	Sensitivity	Overall accuracy
Logistic regression	89.67	0.96	91.67 (0.9167)	96.7 (0.967)	95.1
Naïve bayes	100	0.99	97.5 (0.975)	100 (1)	99.29
Random forest	95	0.89	61.67 (0.6167)	99.09 (0.9909)	89.81

Table 6 shows the prediction performances measure of AUC and etc. According to the above result, each metric depicts that Naïve Bayes performance are best than other models. Naïve Bayes is with highest accuracy. Along with Naïve Bayes, LR also shows immense performance that is 95.10 %. Naive Bayes has most favorable precision (100 %), sensitivity (97.5 %), specificity (100 %), accuracy (99.29), AUC(0.99) and likelihood positive and negative ratios (40,0).Logistic Regression results were also similar to recognize frozen shoulder with sensitivity (91.67), specificity (97.7), precision (89.67), accuracy (95.1), AUC (0.96) and likelihood positive and negative ratios (11,0.04).The predictive data mining models Naïve Bayes, Logistic Regression has statistically better performances than the Random Forest.

In end we adopted the area under the receiver operating characteristics (AUROC). AUROC was used to analyze the biasness, which is basically used to differentiate those who have an Intact or who don’t. Mostly the predictive models accuracy is explained by the area under the curve. We can classify the accuracy of predictive models by matching the points. If result shows value (0.90–1) then the diagnostic test is excellent, for good its value is (0.80–0.90). Table 6 shows the predictive models along with their AUC value.

The areas under the curve for Naïve bayes is 0.99 and for logistic regression its 0.96 and for random forest its 0.89. Overall values shows this Naïve Bayes would be considered to be excellent for separating intact from no-intact group.

The study conclude this Naïve Bayes performed more accurately in TPR (Intact class and the area under curve value is = 0.99) compared to random forest TPR (Intact class area under the curve 0.89).These differences are statistically different.

Models prediction power can be judged through likelihood ratio that’s why we have summarized the likelihood ratios in Table 7. Table 7 is showing likelihood ratio.

**Table 7:** Likelihood ratios

Model	LR+	LR−
Logistic regression	11.60	0.04
Naïve Bayes	40	0
Random forest	2.58	0.01

## Discussion and conclusion

There are many reasons for musculoskeletal disability. After low back pain and neck pain, the third most common cause of musculoskeletal disability is shoulder pain. Frozen shoulder is characterized by pain, stiffness and limited range of motion in shoulder joint. Frozen shoulder condition cannot be identified through X-ray; MRI is carried out to explore this condition. The first description of primary frozen shoulder has been made 150 year ago, but only 16 limited researches has been done to diagnose the underlying cause. According to the cause’s symptoms, secondary frozen shoulder problem may occur after trauma or surgery. Little work has been made on the valuable treatment options for frozen shoulder. Due to limited knowledge about the treatment plan in literature, it’s really tough for diagnosis of the exact problem and recommendation of best treatment plan. All the treatment plans which are mentioned in a literature have a different success rates. Its estimated value in common population is 2–3 % and a 5–6 % ratio is mentioned by the orthopedics surgeons (Mitchell et al. 2005).

This study also verifies that the frozen shoulder problem mostly occurred in female patients. In this research our anticipated, predictive procedures performed well than the statistical techniques. The specificity and sensitivity ratio of an intelligently predicting frozen shoulder are better in the Naïve Bayes and Logistic Regression models as compared to Random Forest. Our results are similar to recent researches which shows that, mining procedures are more helpful than statistical to accurately diagnose different diseases (Wahbeh et al. 2011; Grossi et al. 2007). However, mining techniques has not been used so much by orthopedic doctors for disease prediction. This limitation enlightens that little researches and studies have been done on a predictive data mining in the field of orthopedic. Numerous orthopedic researches and studies have examined the pathology, treatment and capability of the clinical exam to properly identify frozen shoulder. Previous researches also show that there is extensive value of sensitivity (up to 20–91 %) and specificity (up to 20–100 %) for disease diagnosing by Arthography method. That is why then arthography results are compared with sonographic signs (Ryu et al. 1993), but no studies has examined the physical examination variable for disease diagnosing. We established that our predictive mining techniques (Naïve Bayesian and the Logistic Regression) are correct for identifying frozen shoulder. These models having specificity is 91–98 %, and sensitivity is 96–100 %, which is better than earlier researches. If the research shows high sensitivity and moderate specificity, then we can easily use the mining techniques more than statistical for identifying frozen shoulder. We can reduce the unnecessary imaging test ratio and also decrease the false positive rate by data mining procedures.

All the previous studies related to medical statistics were planned to explore the analysis among group data. If requirement is to do medical diagnosis at a single level, then we cannot use the same procedure due to its limitations (Grossi et al. 2007; Chang 2006). During statistical analysis, researchers do a study on a collection of population to disclose relationships among them. Through evidence-based statistics, we can easily predict a diagnosis at an individual level. Therefore, in our study we used the intelligent data mining model, to give an answer at the specific level of classification (Grossi et al. 2007; Chang 2006; Rygielski et al. 2002).

We have developed an intelligent system by using clinical reports, which can be applied to every individual patient of frozen shoulder. During clinical assessments mostly doctors make diagnosis and recommend treatment on the basis of clinical examination and imaging tests. During physical assessment, doctors frequently face different type of problems. On this basis, it’s really difficult for them to determine the disease probability. The classification output of predictive mining was mostly expressed by group method. In this, each individual patient was marked just into one group without knowing that the patients are in the exact class or not. Then, we evaluated the P value against each variable. On the basis of P value results, we have ignored some irrelevant variable. After P value calculation, we used significant variables in a model to predict results and also measure model accuracy. To support our predictive results, we calculated the probability of the prediction outcome with LR (Sox and Harold 1996; Dujardin et al. 1994; Sox et al. 1988) and Fagan’s theorem (Deeks and Altman 2004; Lang and Secic 1997). The sensitivity and specificity of the Naïve Bayesian model were 100 and 97.5 % (as showed in Table 6) which has given a positive LR (LR+) of 40 and a negative LR (LR−) of 0. The sensitivity and specificity of the Logistic Regression model were 96.7  and 91.67 % (as showed in Table 6) which has given a positive LR (LR+) of 11 and a negative LR (LR−) of 0.04 (Table 7) (Deeks and Altman 2004; Lang and Secic 1997; Lu et al. 2014).

The positive likelihood result showed that, if a patient visits a hospital with a frozen shoulder there is 11 % time more chance that it has a positive test than those who doesn’t. On the other hand, if a patient visits without a diagnosed case of a frozen shoulder, then there is approximately 5 % times more chances that its original test will be negative. Previous studies has recommended that if any model shows its LR+ value greater than ten, there are chances that its original test would be positive. On the other hand, if model results show it LR− value less than 1, there are chances that original test would be negative (Akobeng 2007).

In our study, firstly we calculated the predictive model results and also calculated the likelihood ratios. At the end we have used a Bayesian standard theorem, to evaluate the possibility of presence or absent of a frozen shoulder using a prediction result (intact or no-intact) and a prior probability. On the basis of likelihood ratio, doctors can easily give a rough estimate either a patient has a severe frozen shoulder or not. This will also help to tell the severity of disease before recommending an image test (Espallardo 2003). In Bayesian theorem, the main purpose of likelihood ratio is to change the posterior possibility of having disease, after an outcome is well-known (Gill et al. 2005).

We can easily predict the posterior probability by Fagan nomogram. nomo-gram represents usually through graph, that gives the posterior ratio that a disease is exist or not on the basis of predictive results and prior ratio (Akobeng 2007). Fagan nomo-gram tool mark the prior probability on a left side, (a straight line start with a prior ratio of having a disease) and then moved towards the likelihood ratio and at the last intersect at the posterior ration to show ratio of having a disease. For example, if the disease prevalence ratio for a patient is 76 %, according to our Logistic Regression model the patient should be in a class of “intact” with an LR+ 11 (Table 7), Firstly, tool marked the prior probability ratio at the left side which is 76 % then draw a straight line (Fig. 3) and marked the point of LR+ 11 % that intersect at the posterior probability of 97 % (Halkin et al. 1998). This result shows that, when the mining model result is “Intact” then the ratio of having a frozen shoulder for this patient increase from 76 to 97 %.Conversely, when the model outcome is “no-intact” then the possibility of having the patient an intact decrease from 76 to 11 %. Hence, our model outcome help doctors in making diagnostic decision and treatment, mainly if the prior possibility ratio of a frozen shoulder is at the middle. Our model outcomes can be utilized not only to categorize a patient into the ‘‘intact’’ or ‘‘no-intact’’ class but also to change the prior possibility in a way to approximate the posterior possibility.

**Fig. 4:**
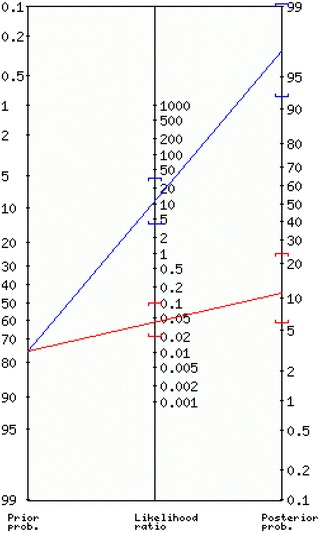
Fagan’s nomo-gram

There are also some limitations for which future research is recommended. First, to deal with the problem which can be occurred due to sample size and also more characteristics of patients are required to deal with the generalization issue (Lawrence and Giles 2000). Secondly, more mining models such as SVM (support vector machine) or decision tree/neural network should also be used to discover improvement of the prediction. Thirdly, more research is needed to examine whether other medical parameters like Capsular Pattern test or the Lift-off test can be good attributes in affecting the miming model performances. Our research did not focus on a surgery or arthroscopy as main principles, although, the real finding can only be guessed by surgery results. In our study, we used MRI results as a reference standard. Instead of this, the performance and legitimacy of our prediction mining models should be further checked on those patients, who are undergoing for a surgery. Figure 4 shows the Fagan’s nomo-gram mapping.

To our information, this study is the initial analysis on an unstructured data with numerous characteristics (age, gender) and disease information (such as disease related, work related), which potentially help the diagnosis of frozen shoulder.
